# Men in Macau SAR have higher prevalence in metabolic syndrome and among related metabolic components: a cross-sectional Macau Health Survey

**DOI:** 10.1186/1471-2458-14-1065

**Published:** 2014-10-11

**Authors:** Tanja Sobko, Dulce Trindade, Qian Xiang Lao, Martin Wong, Tong Ka Io, Chan Kin Wa, Ken Gu

**Affiliations:** Institute of Human Performance, The University of Hong Kong, Room 301, 3/F, 5 Sassoon Rd, Hong Kong, SAR, China; Association of Macau Portuguese Speaking Physicians, Macau Special Administrative Region (SAR), Macau, China; School of Public Health and Primary Care, Faculty of medicine, The Chinese University of Hong Kong, Hong Kong, SAR, China; Laboratory of Civic and Municipal Affairs Bureau, Macau, SAR, China; Health Sciences School, Macau Polytechnic Institute, Macau, Special Administrative Region (SAR), China

**Keywords:** Overweight, Obesity, Cardio-metabolic risk factors, South China, Macau SAR

## Abstract

**Background:**

Macau has recently experienced expansive socioeconomic growth, leading to lifestyle changes that could have contributed to the development of certain diseases. Little information exists on the prevalence of metabolic syndrome (MetS) and associated risk factors. This information is important, since the management of MetS is tightly connected with prevention of cardiovascular diseases in the population.

**Methods:**

This study is based on the cross-sectional Macau Health Survey 2006. Information on anthropometry, physical measurements, socio-demographics, laboratory tests and life-style habits was collected by trained health professionals from a random sub-population sample, aged 18-44 (32.6 ± 8.3). Body Mass Index (BMI) cut-offs were based on WHO criteria for Asian population. The prevalence of MetS, as defined by the International Diabetes Federation was calculated and the associated lifestyle factors were analysed.

**Results:**

Among Macau’s adults (n = 1592), the age-adjusted prevalence of MetS was over two times higher in men (10.5%) than in woman (3.7%), (p <0.01). 15.8% were overweight (BMI ≥23 < 25) and 18.8% were obese (BMI ≥25). Man had significantly higher risk profile in almost all components of MetS (p <0.001), except the waist circumference and HDL. BMI, age and education were significantly related to MetS in both genders (p <0.001).

**Conclusions:**

We found significant gender differences in MetS among the 18 – 44 year old population of Macau, which should be addressed separately in the gender-specific preventive strategies.

## Background

China, due to its large geographical area, varies substantially in demographic characteristics, cultural behaviours and lifestyle habits. This may result in different disease prevalence across the regions, especially those tightly connected with lifestyle behaviours. Metabolic syndrome (MetS) and its components, has been strongly associated with diabetes and cardiovascular mortality [1]. A recent study found gender differences in the prevalence and development of MetS in Chinese population with abdominal obesity, and that men have a more complex profile compared to women [2]. Modification of relevant lifestyle-related risk factors seems to be utterly important to prevent MetS, but the current situation in different parts of China is still unclear. A nationwide survey has shown that the prevalence of obesity in northern China was two-fold higher than that in southern China [3]. Another recent study in South China indicated that a large proportion of Chinese adults have the MetS and associated risk factors, emphasizing the urgent need to develop local strategies for its detection and management [4]. South China is not homogenous: Macau Special Administrative Region (Macau SAR) is a former Portuguese colony, with sovereignty returned to China in 1999, known today as the Las Vegas of Asia. The territory has undergone a rapid economic transition from a less industrialized area to a major city in the region. It has a population of 520 000 (95% of Chinese, 2% Portuguese and 1% Filipinos and 2% of other ethnicities). The information on prevalence of MetS in Macau is lacking. We therefore aimed to estimate the regional prevalence of MetS and associated factors among the population of Macau (18-44 years of age). This particular age was chosen as it represents a possible target for future health promotion in families.

## Methods

### Ethics statement

This study was approved by the Office for Personal Data Protection of the Macau SAR (Dnr SAAG 1719-14/07/2005). Each participant’s written consent was obtained after a full explanation of the study, under the supervision of CDC, Health Bureau, Macau SAR. All data were kept in strict confidentiality and only designated research team members had access to personal information. The authors of this manuscript have certified that they comply with the Principles of Ethical Publishing [5].

### Study population, data collection and survey instruments

This study is based on the first regional cross-sectional population health survey in Macau conducted in 2006 (MHS2006), which adopted a household-based stratified sampling design [6]. The household survey included questions on demography, socio-economic status, medical history and lifestyle. The measurement procedures followed the practice of the U.S. National Health and Nutrition Examination Survey III (NHANES) [7], Personal Well being Index (PWI) [8], and the Hong Kong Health Survey 2003 [9].

From 4189 computer-based randomly selected households, all members aged 18 or above were invited to participate from which 3120 participants were successfully recruited. From the subgroup of 1592, who were (younger than 45 years old), 999 were females (33.0 ± 8.3 years old) and 593 were males (31.9 ± 8.4 years old). Since the sample distribution slightly differed from Macau total population, age adjustment was performed for all prevalence figures with the age group 18-44 years. Education level was classified as college+, middle school, primary & below, whilst employment status was classified as unemployed, full or part time, students, other. Individual and family monthly incomes were recorded in Macau local currency (MOP) and for comparison reasons presented in US dollars (USD). Fasting blood samples were collected at the Health Centres and sent thereafter to Centro Hospitalar Conde De São Januário (CHCSJ) for analysis. Anthropometric, blood pressure and other measurements were performed by trained personnel using standard procedures and calibrated equipment. Finally, face-to-face interviews were conducted by trained interviewers using a structured questionnaires on demographic data, health status and health behaviour. The questionnaires to suit the situation and the purpose of the present study in Macau were adopted based on the US NHANES questionnaire [7] and the Canada National Population Health Survey questionnaire [10]. To test the applicability of the questionnaires and the fieldwork procedures, a pilot survey covering 100 randomly selected Macau residents was conducted prior to this study. The characteristics of the study’s sub-population, stratified by gender are presented in Table 1 and some data on the whole Macau Health Survey 2006 has been presented elsewhere [6].

**Table 1:** **Characteristics of the study population, Macau men and women, aged 18-44 years**

Variables		Woman (n =999) Mean ± SD	Men (n =593) Mean ± SD	P-value
**Age (years)**		33.0 ± 8.3	31.9 ± 8.4	0.019
**Body Mass Index, BMI (kg/m** ^**2**^ **)**		21.4 ± 3.4	23.1 ± 3.7	<0.001
**Waist circumference (cm)**		74.5 ± 8.7	82.6 ± 9.8	<0.001
		**No (%)**	**No (%)**	
**Marital status**	Single	386 (38.6)	248 (41.8)	0.261
	Married	582 (58.2)	335 (56.5)	
	Divorce	17 (1.8)	9 (1.5)	
	Other	14 (1.4)	1 (0.2)	
**Education**	College +	302 (30.2)	186 (31.4)	0.839
	Middle school	583 (58.4)	345 (58.2)	
	Primary & below	114 (11.4)	62 (10.4)	
**Current occupation**	Unemployed	166 (16.6)	50 (8.4)	<0.001
	Full & part time	748 (74.9)	486 (82.0)	
	Student	65 (6.5)	48 (8.1)	
	Other	20 (2.0)	9 (1.5)	
**Income, individual (USD)**	< 500	261 (27.9)	67 (12.2)	<0.001
**Monthly**	501-1000	290 (31.0)	137 (25.0)	
	1001-1500	187 (20.0)	136 (24.8)	
	> 1500	198 (21.2)	208 (38.0)	
**Income, family (USD)**	< 1000	228 (22.9)	93 (15.7)	<0.001
**Monthly**	1001-2500	454 (45.5)	264 (44.5)	
	>2500	315 (31.6)	236 (39.8)	
**Alcohol consumers**	Yes	420 (42.0)	399 (67.3)	<0.001
	No	579 (58.0)	194 (32.7)	
**Exercise**	No exercise	345 (34.5)	167 (28.2)	<0.001
	Mild	603 (60.4)	400 (67.5)	0.005
	Strenuous	51 (5.1)	26 (4.4)	0.517

### Obesity, metabolic syndrome and cardiovascular risk factors definitions

Body mass index (BMI) was calculated as weight (kg) divided by height squared (m^2^) and used to define weight status according to the WHO adult BMI standard adjusted for Asia, BMI ≥23kg/m^2^ < 25 kg/kg/m^2^ for overweight, and BMI ≥25 kg/kg/m^2^ for obesity [11]. The criteria for metabolic syndrome (MetS) were based on the International Diabetes Federation criteria (IDF), which considers the ethnic difference for central obesity: central obesity (waist circumference >90 cm for men and >80 cm for women) plus any other two abnormalities: 1) Hypertension: systolic blood pressure ≥130 mmHg, or diastolic blood pressure ≥85 mmHg, or treatment of previously diagnosed hypertension; 2) Hypertriglyceridemia: ≥1.7 mmol/l or receiving specific medical treatment for this lipid abnormality; 3) Low HDL-cholesterol: <1.03 mmol/l for males or <1.29 mmol/l for females; 4) Elevated fasting blood glucose: overnight ≥5.6 mmol/l [12, 13].

Blood pressure was measured twice with at least 10 minutes of rest prior to the first and second measurement, and the mean values of systolic blood pressure (SBP) and diastolic blood pressure (DBP) were calculated. The procedure followed the Canadian Heart Health Survey recommendations based on the British Hypertension Society protocol [10]. Participants were seated in a chair with the back, arms supported and the elbow crease of the right arm was positioned at the apex of heart with the palm facing down.

Based on the recommendation from the American Heart Association [14], dyslipidaemia was defined as high total cholesterol (CHOL) ≥6.15 mmol/l, high low-density lipoprotein cholesterol (LDL-C) ≥4.1 mmol/l, low high-density lipoprotein cholesterol (HDL-C) <1.28 mmol/l in females or <1.03 mmol/l in males or hypertriglyceridemia (TG) ≥2.25 mmol/l. Diabetes mellitus was defined as self-reported diabetes and high fasting plasma glucose ≥7.0 mmol/l.

### Lifestyle factors: exercise and alcohol consumption

Physical activity was estimated from the questionnaire [15], while self-reported alcohol consumption was calculated from the usual daily intake of alcoholic beverages. Frequency and extent of physical activity was reported on a weekly basis, including sports, physically active hobbies, and fitness exercises over the past 30 days. Exercise was defined as any activity for at least 30 min in preceding 3 months, causing light perspiration or a slight to moderate increase in heart rate or in breathing. The International Physical Activity Questionnaire (IPAQ) – long version [15] was used to categorize participation in physical activity into 3 groups: no exercise, mild and strenuous.

#### Statistical analysis

Categorical variables were expressed as percentage and numerical variables were expressed as mean and standard deviation (SD). Most of these variables were further stratified by age and sex. All outcome variables were tested for normality and non-parametric tests were used for not normally distributed variables. Differences between two groups in continuous variables were tested using t-test or Mann–Whitney–Wilcoxon test; differences between two categorical variables were tested using Chi-square. The correlation analyses (Pearson or Spearman correlation) were performed to test the relationship between two continuous variables. Bi-variate analysis tested MetS vs each of the other variables. To examine some important determinants of the MetS among demographic anthropometric and lifestyle factors, a multiple logistic regression analysis was performed. MetS was a dependent variable and independent variables were sex, education, marital status, employment, individual and family income, BMI, alcohol consumption and exercise. The rate ratios (RR) are presented together with 95% CI. All tests were two-tailed and p-values <0.05 were regarded as statistically significant. All data was analysed by SPSS version 17.0.

## Results

### Prevalence of the metabolic syndrome and metabolic components

Among the study participants, 57.4% were married (58.2% women and 56.5% men). The majority, 58.3%, had middle school education (58.4% women and 58.2% men) and 79.5% had full or part time jobs (74.9% women and 82.0% men). The unemployment rate was significantly higher in females than in males (16.6% vs 8.4%), (p < 0.01). Additionally, more men than women had a higher individual income (USD 501+ per month; 87.8% vs 72.2%); 21.2% women and 38% men earned more than 1500 USD per month (Table 1).

**Figure 1:**
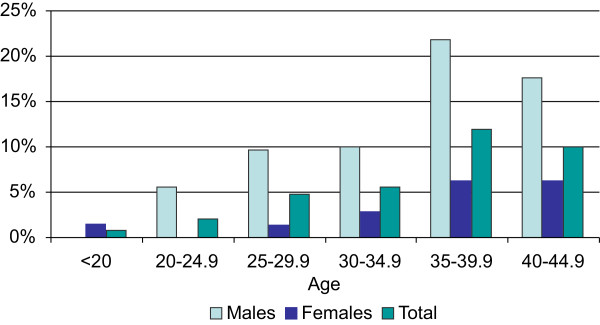
**Prevalence of metabolic syndrome (MetS) based on IDF criteria, stratified by age and sex.**

In the adult population of Macau (18 – 44 years old), the age-adjusted prevalence of MetS was significantly higher in men than in women 10.5% and 3.7% respectively. The prevalence reached over 20% in men during the age range of 35 – 39.9 years (p < 0.001), (Figure 1, Table 2). 15.8% were overweight (BMI ≥23 < 25) and 18.8% were obese (BMI ≥25). Significantly more men than women, aged 25 – 44 years, were overweight and obese, (p <0.01). The different components of MetS are presented in the Table 2. The abdominal obesity did not differ, 24.2% in women and 21.9% in men. Prevalence of high fasting glucose was doubled among men compared to women, 9% and 4.3% respectively (p <0.001). Significantly more men than women had high blood pressure (BP >130/80 mmHg), 36.6% vs. 8.6% (p <0.001). The rate for hypertriglyceridemia (>1.7 mmol/l) was 5.8% in women and 26.7% in men, (p < 0.001) and men had significantly higher risk to have hypercholesterolemia than women (OR = 1.75, p < 0.001). High cholesterol levels increased with age in both men and women. Overall, men had dramatically higher prevalence in almost all metabolic components, except the waist circumference and HDL.

**Table 2:** **Sex-stratified prevalence of each component of metabolic syndrome (MetS) and clustering of the components, International Diabetes Federation criteria (IDF)**

Variables	Women (%, n =999)	Men (%, n = 593)	P value
Abdominal obesity	24.2	21.9	0.294
Systolic Blood Pressure, SBP ≥130 mmHg	8.4	33.9	<0.001
Diastolic Blood Pressure, DBP ≥85 mmHg	3.1	16.7	<0.001
SBP ≥ 130 mmHg and DBP ≥85 mmHg	8.6	36.6	<0.001
High SBP or high DBP	9.3	37.8	<0.001
Hypertriglyceridemia, ≥1.7 mmol/L	5.8	26.7	<0.001
Low HDL C	6.5	4.6	0.106
Fasting blood glucose ≥5.6mmol/L	4.3	9.0	<0.001
Fasting blood glucose	5.0	9.1	0.001
Prevalence of MetS, age-adjusted	3.7	10.5	<0.001

### Associated factors for metabolic syndrome

Associations between demographic, socioeconomic, anthropometric, some lifestyle factors and the risk for the MetS are shown in the Tables 3 and 4 respectively for women and men. RR of lowest education level (primary & below’) for MetS was 2.1 (4.2, 10.1) in men and 7.2 (1.9, 26.6) in women (Tables 3 and 4). Overall, one third of the participants (31.4%) had basically no exercise (less than once a month), p <0.001, 64% were actively engaged in mild exercise, 3 times or more a week p =0.005, and 4.8% had reported strenuous exercise, p =0.517, (Table 1).

**Table 3:** **Rate ratios (RR) for metabolic syndrome (MetS) as a dependent variable and associated factors as independent variables among Macau women, aged 18-44 years (n =999)**

Variables	Categories	% with metabolic syndrome	Rate ratio (95% CI)	P value
**Education**	College+	1.00	1.0	
	Middle School	4.50	4.5 (1.4, 14.7)	0.01
	Primary & below	7.10	7.2 (1.9, 26.6)	< 0.001
**Employment**	Unemployed	6.70	1.0	
	Full or part time	3.30	0.5 (0.3, 0.9)	0.05
	Students	1.50	0.2 (0.03, 1.7)	0.11
**Individual**	>1500	2.50	1.0	
**Incomes**	1001-1500	5.50	2.2 (0.8, 5.9)	0.11
**USD/month**	501-1000	1.60	0.6 (0.2, 2.6)	0.53
	<500	4.60	1.8 (0.7, 5.1)	0.24
**Family**	>2500	1.90	1.0	
**Incomes**	1000-2500	4.00	2.1 (0.8, 5.2)	0.11
**USD/month**	<1000	5.70	3.0 (1.2, 7.8)	0.02
**Exercise**	No exercise	2.90	1.0	
	Mild	4.30	1.5 (0.7, 3.1)	0.27
	Strenuous	2.20	0.7 (0.1, 5.2)	0.70
**Alcohol consumption**	Yes	3.3	1.0	
	No	4.0	1.2 (0.6, 2.3)	0.58
**Body Mass Index (kg/m** ^**2**^ **)**	<18.5	0.00		
	18.5-22.9	1.30	1.0	
	23-24.9	4.80	3.8 (1.3, 11.2)	0.01
	25-26.9	12.90	10.3 (4.0, 26.7)	<0.0001
	27+	23.10	18.4 (7.8, 43.5)	<0.0001

**Table 4:** **Rate ratios (RR) for metabolic syndrome (MetS) as a dependent variable and associated factors as independent variables among Macau men aged 18-44, (n =593)**

Variables	Categories	% with metabolic syndrome	Rate ratio (95% CI)	P value
**Education**	College+	8.6	1	
	Middle School	10.1	1.2 (0.7, 2.1)	0.57
	Primary & below	17.7	2.1 (4.2, 10.1)	0.05
**Employment**	Unemployed	8.3	1	
	Full or part time	11.7	1.4 (0.5, 3.7)	0.48
	Students	2.1	0.3 (0.03, 2.2)	0.17
**Individual**	>1500	12.0	1.0	
**Incomes**	1001-1500	16.8	1.4 (0.8, 2.4)	0.21
**USD/month**	501-1000	7.4	0.6 (0.3, 1.2)	0.16
	<500	4.5	0.4 (0.2, 1.2)	0.08
**Family**	>2500	7.6	1.0	
**Incomes**	1000-2500	12.1	1.6 (0.9, 2.8)	0.09
**USD/month**	<1000	12.9	1.7 (0.9, 3.4)	0.13
**Exercise**	No exercise	15.6	1.0	
	Mild	8.3	0.5 (0.3, 0.9)	0.01
	Strenuous	11.5	0.7 (0.2, 2.3)	0.59
**Alcohol consumption**	Yes	11.3	1.0	
	No	8.0	0.8 (0.4, 1.3)	0.58
**Body Mass Index (kg/m** ^**2**^ **)**	<18.5	1.9	1.0	
	18.5-22.9	0.4	0.2 (0.01, 3.4)	0.23
	23-24.9	1.6	0.9 (0.1, 9.2)	0.89
	25-26.9	18.2	9.8 (1.3, 72.5)	<0.0001
	27+	51.0	27.6 (3.9, 194.7)	<0.0001

Additionally, men, who regularly participated in mild exercise, had a significantly lower RR for MetS 0.5 (0.3, 0.9), (Table 4). Using logistic regression with MetS as dependent variable and Age, BMI, Education, Individual Income, Family Income, Employment Status, Exercise, and Alcohol Consumption as independent variables we found that BMI and age were significantly related to MetS in males and females separately through a forward modelling method. Females in the higher BMI group (25+) had 40% more risk to have MetS compared with the group (<25) (OR = 1.4, 95% CI: 1.2 - 1.5, p = 0.000); with every year, the risk for MetS increased 7.7% (OR = 1.1, 95% CI: 1.004 - 1.155, p = 0.039). Men in higher BMI group (25+) had 90% more risk to have MetS compared with those in the lower BMI group (<25) (OR = 1.9, 95% CI: 1.6 - 2.3, p = 0.000), the risk for MetS increased yearly 10.7% (OR =1.1, 95% CI: 1.028 - 1.192, p = 0.007), (Table 5).

**Table 5:** **Results of logistic regression analysis of risk factors for metabolic syndrome**

		Odds ratio (95% CI)	P value
**Females**	BMI	1.37(1.23-1.53)	.000
	Age	1.08(1.00-1.17)	.041
	Exercise	.17(.02-1.70)	.131
	Education	.61(.20-1.82)	.374
	Employment	1.43(.36-5.69)	.611
	Ind. Income	1.96(.52-7.36)	.319
	Fam. Income	.64(.23-1.79)	.395
	Alcohol use	.51(.20-1.29)	.156
	Constant	.00	.001
**Males**	BMI	1.90(1.58-2.30)	.000
	Age	1.09(1.01-1.18)	.028
	Exercise	1.24(.10-15.89)	.869
	Education	.37(.09-1.51)	.167
	Employment	10.10(.81-125.72)	.072
	Ind. Income	11.58(.73-183.78)	.083
	Fam. Income	1.65(.54-5.01)	.378
	Alcohol use	.59(.19-1.85)	.368
	Constant	.000	.000

BMI: body mass index; 95% CI: 95% confidence interval.

## Discussion

This study examined gender differences in the prevalence of MetS, its components and some associated risk factors among the population of Macau. The age-adjusted prevalence of MetS was significantly higher in men than in women 10.5% and 3.7%, p < 0.001. The situation worsened dramatically during the ages of 35 – 40 years (Figure 1). In total 15.8% were overweight and 18.8% were obese (BMI ≥25). Overall, men had a higher prevalence in almost all of the metabolic components (Table 2). These results for the first time show the true situation in Macau SAR, confirming that MetS, a significant current problem in mainland China [4], has reached Macau. This is in accordance with the theory of thrifty genotype [16], stating that the transformation of a developing region to a developed municipal city might contribute to unhealthy habits, sedentary life style and other determinants of an obesogenic environment [16]. The real per capita GDP of Macau exceeds that of Hong Kong since year 2005, and the territory had been ranked the third in the Asia Pacific [17]. The neighbouring cities, such as Hong Kong, heavily influence Macau’s patterns of food consumption and physical activity habits, replacing the traditional health-enhancing practices. Macau’s booming tourist industry enhances the negative energy balance and sedentary lifestyle.

Lower education in the general population of China had been previously shown to be associated with higher risk of MetS in women, but not in men [18]. Our study shows that such lower education in both gender, and lower income in women are associated with a higher prevalence of MetS. Furthermore, women working full or part time had a lower rate of MetS. This difference seems to be more pronounced in Macau, as the majority of the population is employed in the casinos, where a higher education is not necessary. Additionally, being overweight among men in China, and particularly in Macau, has not been traditionally considered negative, but on the contrary, implies wealth and prosperity. Similar to findings from Korea, by Park et al., our study confirmed BMI being a sensitive modifiable risk factor among the associated risk factors for MetS [19].

The age range of our study population is 18 – 44 years; we particularly addressed the health status of adults of reproductive age, since their status will probably influence habits of the entire family. The main finding of this investigation is that Macau men (current or potential fathers) seem to have a higher risk to develop MetS, especially if they have lower education and are inactive. A recent study has confirmed a significantly increased risk of MetS in adolescents with a MetS parent [20]. Furthermore, a recent study in Sothern China [4] has reported that compared to women, men had a higher waist circumference, diastolic blood pressure, triglycerides, as well as a lower HDL-cholesterol level, although women had a higher prevalence of MetS than males (8.99% vs. 5.27%). These results cover the entire population, while the age in our study is 18-44 years, and may therefore be difficult to compare. Hong Kong survey 2003/2004 reported that overweight and obesity, hypertension, high blood cholesterol and diabetes were among the most prevalent chronic conditions with a higher proportion of males (42.5%) than females (35.9%) [9], but our findings reflect that the gender difference in Macau is much greater. Comparing to the general situation in greater China, the gender difference in the prevalence of different MetS components in Macau population differs from previously published findings. The prevalence Indices are lower and display different pattern. For example, the Inter-ASIA study, which is a national Chinese population sampling survey, indicated hypertension as the most common component of MetS in males (44.2%), whereas in females HDL-C was most common (46.5%) [3]. In our study, the most frequent component of MetS in women was abdominal obesity (24.2%), but in men, was hypertension (36.6%), followed by hypertriglyceridemia (26.7%) and abdominal obesity (21.9%). Findings of the Inter-ASIA study and this current study differ possibly due to the age of the participants. We included adults aged 18-44 years while the age Inter-ASIA study included older participants (35-74 years). We postulate that subgrouping of the population gives more detailed information of this particular age group.

The pattern of MetS components was more complex in males than in females. In contrast to recent research [2], our study did not show any gender difference in abdominal obesity and HDL. However, men had a significantly higher prevalence in the rest of the components. Thus, we find that males with MetS had a higher risk profile than females.

We have demonstrated that men have a higher risk of developing MetS. This gender difference could be partly explained by the younger pre-menopausal age of the women in our subpopulation, as the cardiovascular risk increases markedly after menopause [21]. The prevalence of MetS in pre-menopausal women, significantly lower than that in men, gradually surpasses males after menopause, as described in Hwang et al [22]. This could be the case in our study if women over 45 were included.

Once again, our study confirms the positive effect of exercise; mild level of physical activity seems to be enough. Although the IPAQ questionnaire refers to the last 7 days, the findings regarding the relationship between exercise and MetS in the Tables 3 and 4 should be interpreted with caution, since some people may have started to exercise after the emergence of MetS. Younger men are more physically active, gradually exercise less and catch up the levels of inactivity of females by the age of 35. Although it is commonly recognized that mothers spend more time with children, it is important to further investigate the father’s influence on children’s attitudes. As the study continues, it will be valuable to follow up the overweight fathers and their offspring, particularly boys, to explore the magnitude of this influence.

Some limitations of our study’s regional estimates should be mentioned.

First, the response rate of the MHS2006 is somewhat low. Second, the responded sample of the population might have been more health oriented than the ones who did not respond. Third, the results presented in this report lack information on diet, which is extensive. It is analysed and presented in a separate paper. Furthermore, information on smoking is lacking, it is one of the direct causes of coronary artery disease, indirectly inducing hypertension and diabetes in China [23]. Despite the newly launched National anti-smoking campaign and strict regulations in Macau, a big proportion of local population continues to smoke. Because the information on smoking was collected by a separate self-administrated form, containing other sensitive questions, this made it impossible to trace and analyse the smoking effect on our parameters. Finally, there were more women than men in our study, since females had a relatively higher response rate than males, which might cause a selection bias. Overall, as we determined the prevalence of MetS in Macau, we had difficulty in including more parameters in our logistic regression model, such as family history of hypertention, more detailed information on drinking, smoking etc, because at the times of the data collection, naturally less was known about the potential risk factors. This, in turn, underlines the importance of this report, making the comparison with the current situation possible. The cross-sectional study presented here does not allow us to draw any cause-effect relationship. Therefore, future prospective studies are needed to confirm the association between the suggested here and by others exposures and MetS among the population of Macau.

Despite these limitations, our study represents the first regional prevalence estimate of MetS, overweight and obesity among the adult Macau population. The study findings confirm that MetS continues to be a serious health hazard in this population. Furthermore, most preventive strategies today are general, not taking into consideration the situation locally and we believe that our data will help to fill this gap. This report not only serves as important baseline to track changes over time, but also provides clinicians and public health professionals with useful information essential for policy planning at regional and national levels.

Recent important study in China showed that although the average blood pressure levels and prevalence of hypertension among adults in the rural areas of China had dramatically increased during last decades, the awareness, treatment and control of hypertension remained low [24]. Similar to the suggestions by the authors, our study emphasizes that public health programs should be more specific, prioritizing, for example, prevention and control of hypertension, which will in turn reduce the occurrence of MetS and cardiovascular diseases.

Physical inactivity may enhance risk for MetS and elevate the risk for developing cardiovascular disease in people with MetS [25, 26]. With obvious rapid economic progress, Macau has been experiencing a lifestyle and dietary transition, which brought increasing inactivity and a shift towards high-fat, high-energy-density and low-fiber diet [25, 26]. Our study has also indicated that the prevalence of MetS was positively associated with inactivity. It therefore is logic that the health promotion programmes should focus on improving physical activity, especially in the male population of Macau. Potential interventions should determine, collect and analyze detailed data on the level of and quantity of physical activity among Macau population. Several steps can be taken to reduce the prevalence of potential risk factors contributing to the development of MetS in this population. Apart from dietary modifications, an increased physical activity may have implications to reduce this health burden. Implementation of these promotion strategies require support from the community. Thus, more studies aiming at examining the medical costs associated with MetS are needed. This will further emphasize how critical is the need for education and training of health care providers. Only then they will be able to have the knowledge necessary not only to treat the existent MetS patients but to develop effective MetS prevention programs on the community level.

## Conclusions

In conclusion, we analyzed gender differences in the prevalence of MetS and its components in a Macau SAR population. We found the prevalence of MetS in this population was significantly higher in men than in women, especially with lower education and higher income status. This work lays the ground for understanding the recent obesity increase in the Macau population and is essential for the development of new policies, innovative programs and locally developed prevention strategies. Based on our results concerning gender differences in metabolic disease development, we suggest that emphasis of the programs related to MetS should be focused on early detection of metabolic risk factors, prevention, and interventions that should consist of gender-specific issues when addressing the family.

### What is already known on this subject?

Very little information on the prevalence of metabolic syndrome (MetS) and associated risk factors exists today in this special region of China, where the majority of the population is involved in the casino activities. Furthermore, South China’s population is not homogenous and Macau is a good example, living under the Portuguese influence until recently.

### What this study adds?

Firstly, this is the first representative regional data, which we continue to follow-up, giving the possibility for international comparisons. To gain this information is important, since the management of MetS is tightly connected with prevention of cardiovascular diseases in the population.
